# Huangqi Guizhi Wuwu Decoction Improves Arthritis and Pathological Damage of Heart and Lung in TNF-Tg Mice

**DOI:** 10.3389/fphar.2022.871481

**Published:** 2022-05-04

**Authors:** Yi Wang, Tao Chen, Can Yang, Qiang Li, Mengjiao Ma, Hao Xu, Qi Shi, Yongjun Wang, Youhua Wang, Qianqian Liang

**Affiliations:** ^1^ Longhua Hospital, Shanghai University of Traditional Chinese Medicine, Shanghai, China; ^2^ Spine Institute, Shanghai University of Traditional Chinese Medicine, Shanghai, China; ^3^ Cardiovascular Department, Longhua Hospital, Shanghai University of Traditional Chinese Medicine, Shanghai, China; ^4^ Key Laboratory of Theory and Therapy of Muscles and Bones, Ministry of Education, Shanghai University of Traditional Chinese Medicine, Shanghai, China; ^5^ Central Hospital of Jing’an District, Fudan University, Shanghai, China

**Keywords:** Huangqi Guizhi Wuwu Decoction, rheumatoid arthritis, cardiopulmonary complications of RA, TNF-Tg mice, traditional Chinese medicine

## Abstract

**Background:** Huangqi Guizhi Wuwu Decoction (HGWD) is a traditional and effective Chinese medicine compound decoction for the treatment of rheumatoid arthritis (RA). However, there is few research on the treatment of rheumatoid cardiopulmonary complications. The present study was to study whether HGWD can alleviate the pathological changes caused by rheumatoid arthritis and cardiopulmonary complications.

**Methods:** Five 3-month-old TNF-Tg mice were treated with HGWD (9.1 g/kg) once a day or the same dose of normal saline lasted for 8 weeks, and wild-type littermates of the same age were used as a negative control, and methotrexate (MTX) was intraperitoneally administered as a positive control. After the treatment, pathological staining was performed on the mouse ankle joints, heart, and lungs.

**Result:** It was found that HGWD reduced the inflammation of the ankle joint synovium in TNF-Tg mice, and reduced myocardial hypertrophy, inflammatory infiltration and fibrosis of heart, as well as lung inflammation and fibrosis. Immunohistochemical staining with anti-TNF-*α* antibody showed that HGWD reduced the expression of TNF-*α* in the heart of TNF-Tg mice.

**Conclusion:** In conclusion, HGWD alleviates joint inflammation in TNF-Tg mice and reduces the pathological changes of the heart and lungs.

## Introduction

Rheumatoid arthritis is a chronic autoimmune disease characterized by multi-articular, symmetrical, aggressive joint inflammation of the hands and feet. In addition to joint symptoms, complications of rheumatoid arthritis include interstitial lung disease, osteoporosis, atherosclerosis and rheumatoid vasculitis (Charles-Schoeman, 2012; Xue et al., 2016; Ruaro et al., 2018; Yamakawa et al., 2021) may occur. Interstitial lung disease (ILD) is a common complication of rheumatoid arthritis and threatens the lives of patients with rheumatoid arthritis (Wells and Denton, 2014; Spagnolo et al., 2018). Clinical investigations have shown that the survival rate of patients with RA complicated by interstitial pneumonia is significantly reduced, and the mortality rate of RA-related ILD (RA-ILD) patients is 2–10 times that of non-ILD patients (Hyldgaard et al., 2017; Zamora-Legoff et al., 2017). Cardiac complications also seriously affect the quality of life of patients. A retrospective study of nearly five decades pointed out that the risk of death in patients with RA, 1.5 times increase over the average person (Dadoun et al., 2013), and the mortality rate is attributed in large part to an increase in cardiovascular complications occurs (about 30–40%) (Gabriel, 2008; van den Hoek et al., 2017; Nakajima et al., 2010; England et al., 2018).

To reduce disease activity or relieve symptoms, RA is mainly treated with inflammation control (Sparks, 2019). Currently available drugs include non-steroidal anti-inflammatory drugs, glucocorticoids, and DMARDs of synthetic origin or biological origin. Wherein methotrexate (MTX) is considered appropriate first-line therapy in patients with RA (Burmester and Pope, 2017; Sparks, 2019). However, for RA-ILD is still no clear treatment recommendations on clinical, and the use of MTX in the treatment of RA-ILD is controversial (Fragoulis et al., 2019a; Cassone et al., 2020). Cardiovascular comorbidities in patients with RA can be managed by lowering disease activity and reducing traditional cardiovascular risk factors. But the use of non-steroidal anti-inflammatory drugs and glucocorticoids can increase the risk of cardiovascular complications (van Breukelen-van der Stoep et al., 2013; England et al., 2018).

TNF-Tg mouse is a RA transgenic mouse with chronic and persistent overexpression of human TNF-*α* and pathological manifestations of symmetry and polyarthritis (Chen et al., 2016). TNF-Tg mouse is a slow onset, high incidence, and stable model, and has become a commonly used research model for RA. Richard D Bell (Bell et al., 2019; Wu et al., 2019) found that in addition to RA symptoms, TNF-Tg mice are also complicated by pulmonary interstitial fibrosis and right ventricular hypertrophy. Also, the incidence in the female is higher than that of males, and the cardiopulmonary complications of TNF-Tg mouse will accelerate its death. Therefore, we chose this model animal to study the treatment of RA and its cardiopulmonary complications.

Huangqi Guizhi Wuwu Decoction (HGWD) is a classic prescription of traditional Chinese medicine, which comes from the synopsis of the Golden Chamber written by Zhang Zhongjing in the Eastern Han Dynasty. It consists of *Astragalus mongholicus* Bunge (Huangqi), *Neolitsea cassia* (L.) Kosterm. (Guizhi), *Paeonia lactiflora* Pall*.* (Baishao), *Zingiber officinale* Roscoe (Shengjiang), and *Ziziphus jujuba* Mill. (Dazao). It is commonly used in the treatment of RA, diabetic peripheral neuropathy, cervical spondylosis, coronary atherosclerotic heart disease, angina pectoris and sequelae of cerebral infarction, and others (Li et al., 2014; Han et al., 2013). However, there is no research to explore whether Huangqi Guizhi Wuwu Decoction has a therapeutic effect on the pathological damage of the heart and lungs in rheumatoid arthritis. Therefore, TNF-Tg mice were selected and treated with HGWD, compared with only saline and traditional DMARDs drug MTX. The purpose of this study was to explore the pharmacological effects of HGWD in treating joint inflammation in TNF-Tg mice and its complicated cardiopulmonary diseases.

## Materials and Methods

### Animals

A total of 20 mice were used in this study, consisting of 15 TNF-Tg mice and five littermate controls. The TNF-Tg mice used in the experiment were presented by the University of Rochester, and they were raised at the Shanghai Research Center of the Southern model at a room temperature of 22–24°C, relative humidity of 50–60%, and 12h/12 h under light and dark conditions. They were fed regularly and quantitatively. The research protocols follow the principles of animal care and use. Animal experiments were approved by the Animal Care Committee of Shanghai Research Center of the Southern model (2018-0026).

### Herbal Ingredients and Preparation

HGWD consists of *Astragalus mongholicus* Bunge (Huangqi) 9g, *Neolitsea cassia* (L.) Kosterm*.* (Guizhi) 9g, *Paeonia lactiflora* Pall*.* (Baishao) 9g, *Zingiber officinale* Roscoe (Shengjiang) 18g, and *Ziziphus jujuba* Mill. (Dazao) 15 g. The drugs were purchased from the Chinese Pharmacy of Longhua Hospital Affiliated with the Shanghai University of Traditional Chinese Medicine. The concentration of HGWD taken by mice is 0.91 g/ml. The above-mentioned Chinese herbal medicines were soaked in water for 1 h, boiled twice and the drug was filtered, and the two soups were combined, concentrated to 66 ml by a rotary evaporator, and stored at −20°C. The active components in HGWD have been verified by HPLC quality control (Supplementary Material).

### Treatment

Five three-month-old TNF-Tg mice were given HGWD (9.1 g/kg) once a day, 0.2 ml each time, or given the same amount of normal saline for 8 weeks. Nontransgenic littermates are used as aged-matched wild-type (WT) controls. In the MTX group, intragastric methotrexate was used at the dose of 0.15 mg/ml as a positive control, intraperitoneal injection of 0.1 ml each time, twice a week.

### Histology

Use 4% paraformaldehyde to fix the mouse ankle joints, heart, and lungs for 48 h. The ankle joint was decalcified with 10% EDTA for 21 days and then embedded in paraffin after dehydration treatment with a dehydrator. The heart and lungs were embedded in paraffin after dehydration. The thickness of each slice is 4 um. Hematoxylin and eosin (HE) staining and Masson staining were used to observe histopathological changes. Used Olympus VS120-SL to take slices and Image J was used to measure the area of inflammation and fibrosis.

### Immunohistochemistry

The myocardial cell membrane was stained with WGA antibody (ZF0305, Vector Laboratories), diluted with 5% BSA at a ratio of 1:500, incubated for 2 h at room temperature, washed with PBS 1X, and then mounted with DAPI anti-fluorescence quenching mounts, and collected by a microscope.

The paraffin sections of the heart were deparaffinized. According to the instructions of the reagents, used 1% H_2_O_2_ for 10 min, Used sodium citrate repair solution (P0083, Beyotime) to repair antigen in a microwave oven, and 5% BSA for 30 min. Afterward, incubate with TNF-*α* primary antibody (AF-410-SP, R&D) at 20 ug/ml at 4°C overnight. After washing with PBS 1X, add anti-goat secondary antibody and incubate for 30 min. SABC reacted for 30 min, washed and stained with DAB staining solution, hematoxylin stained the nucleus, and then mounted the slide and took pictures under a microscope.

### Statistical Analysis

Statistical images are generated in GraphPad prism 8.4. The data were analyzed using SPSS26., and the experimental data are expressed as means ± standard deviation. When the homogeneity of variance test and the normality test are satisfied, the One-way ANOVA test followed by the Bonferroni posttest was used for multiple group comparisons. The inspection level is *α* = 0.05, statistically significant differences were considered when *p* < 0.05.

## Result

### Huangqi Guizhi Wuwu Decoction Reduces the Severity of Arthritis in TNF-Tg Mice

**FIGURE 1:**
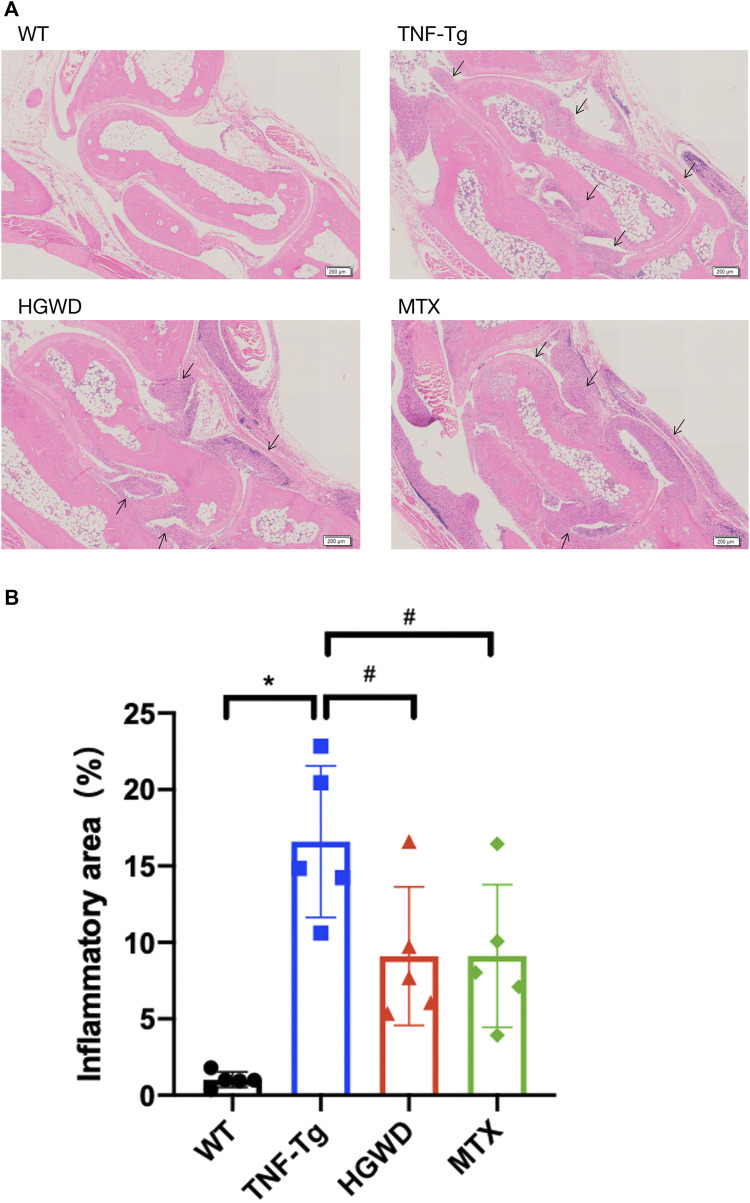
HGWD alleviates the infiltration of ankle joint inflammation in TNF-Tg mice. The treatment group was gavage with HGWD, MTX was administered as the positive control group and nontransgenic littermates were used as the wild-type control group. After administration, the optimal right ankle section of each mouse was used for HE staining, and a slice was selected from each mouse as sample statistics. **(A)** Representative HE stained sections show that the inflammation of the ankle joint of HGWD-treated mice was reduced. Bar = 200 μm, the black arrow indicates inflammatory synovial tissue. **(B)** Quantification of synovial area. The values are the mean plus or minus SD of each group of 5 legs. *p < 0.05, compared with WT group; ^#^ *p* < 0.05, compared with TNF-Tg group.

In order to observe the therapeutic effect of HGWD on arthritis in TNF-Tg mice, we used HE staining to stain the right ankle joints of mice after 8 weeks of intragastric administration of HGWD and quantified the area of inflammation in the ankle joint synovium. We found that the ankle joints of TNF-Tg mice had severe synovial inflammation infiltration compared with WT mice (Figure 1A). Compared to the saline group, administration of HGWD can significantly improve synovitis of TNF-Tg mice, which was no significant difference compared with the MTX group (Figures 1A,B).

### Huangqi Guizhi Wuwu Decoction Reduces Cardiac Hypertrophy, Cardiac Inflammatory Infiltration, and Fibrosis in TNF-Tg Mice

**FIGURE 2:**
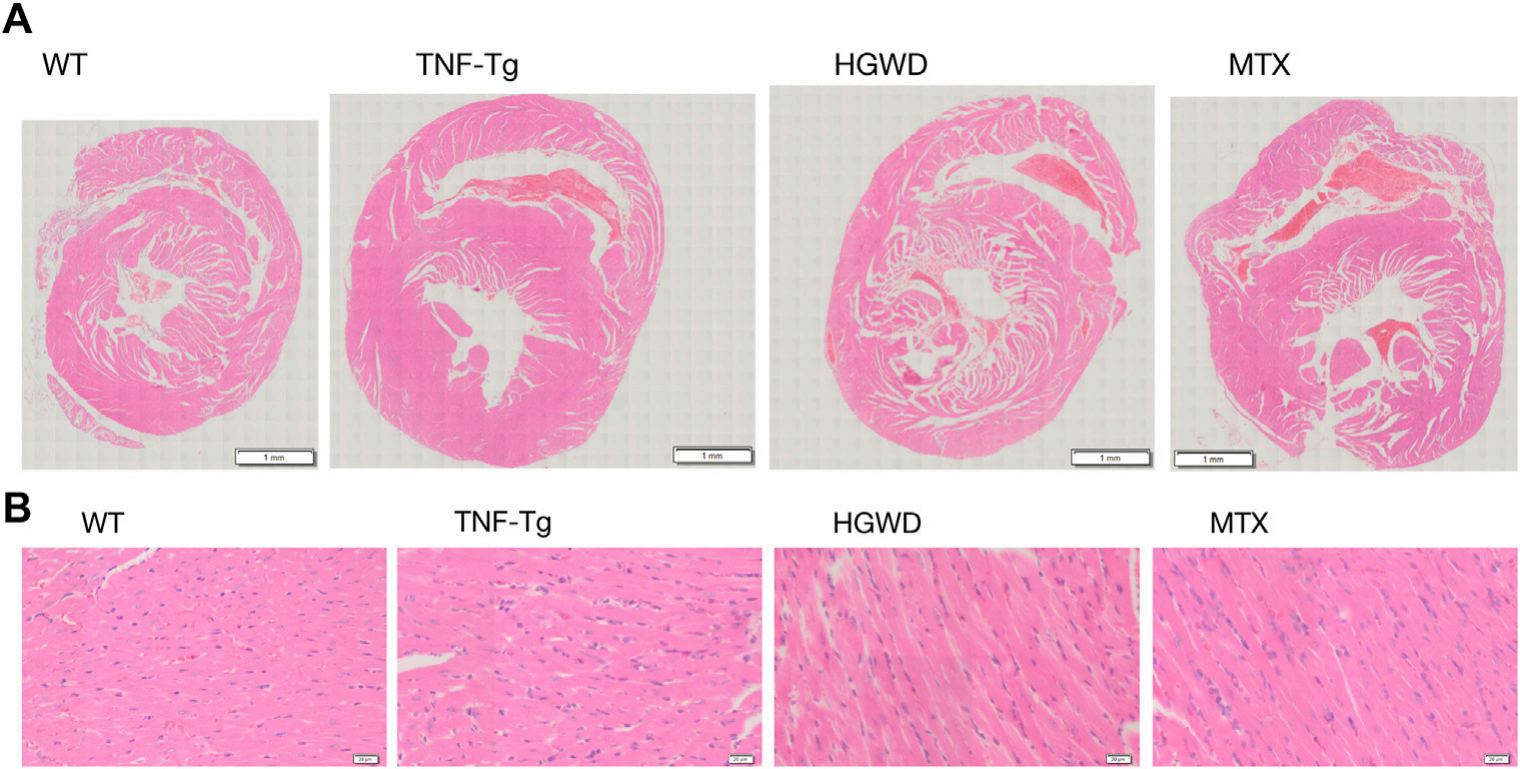
HGWD reduces the inflammatory infiltration of the heart of TNF-Tg mice. Mouse hearts were taken for HE staining analysis. **(A)** Representative HE stained sections show that the heart of TNF-Tg mice has obvious inflammation infiltration, and the arrangement of cardiomyocytes is disordered. The inflammatory infiltration of the heart of mice treated with HGWD was reduced. **(B)** Partially enlarged picture.

To observe the therapeutic effect of Huangqi Guizhi Wuwu Decoction on myocardial injury in TNF-Tg mice, we performed HE staining on the mouse heart and found that compared with the WT group, the myocardial tissue of TNF-Tg mice had obvious inflammation infiltration, and the cell arrangement was disordered (Figures 2A,B).

**FIGURE 3:**
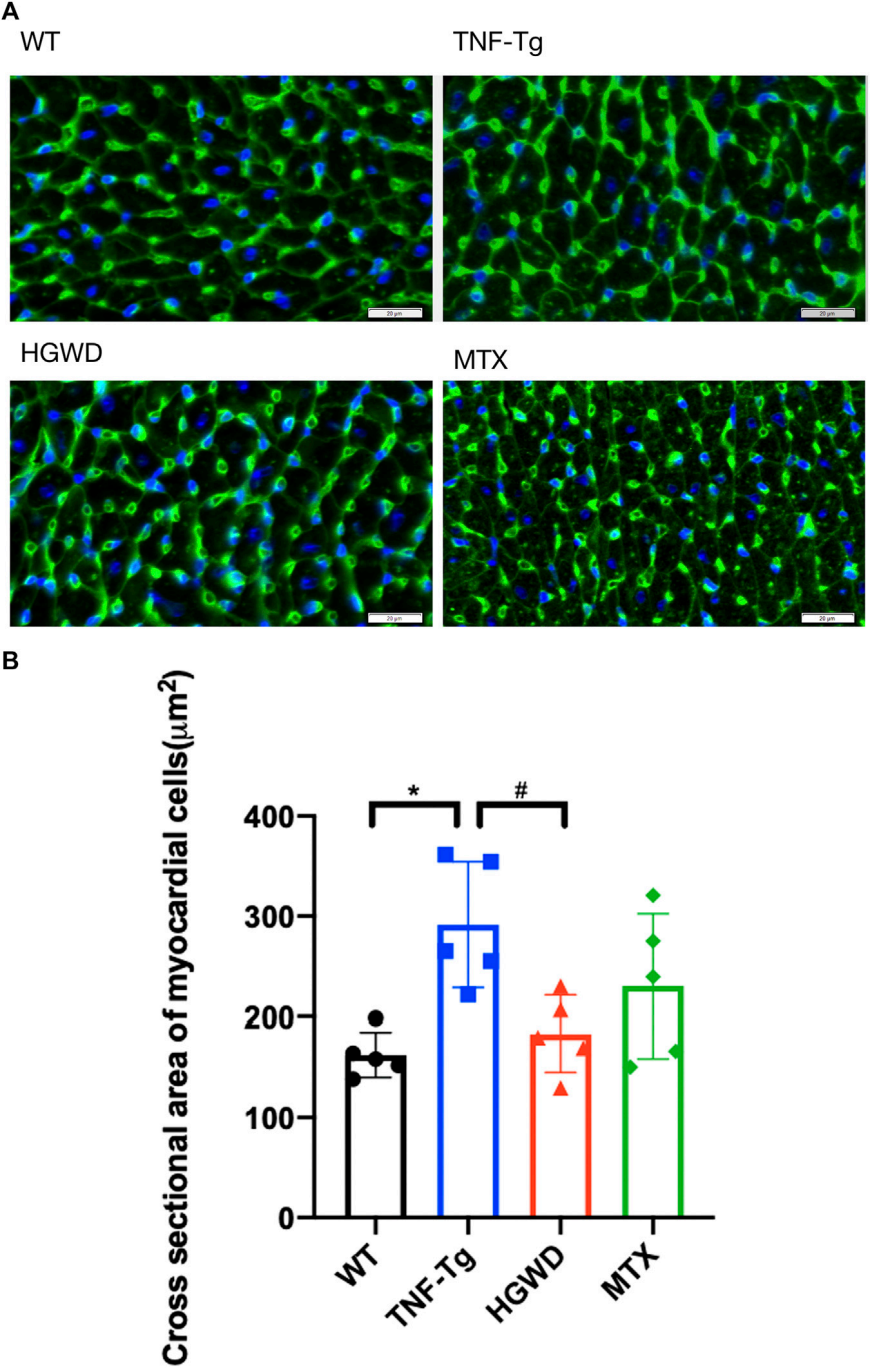
HGWD alleviates cardiomyocyte hypertrophy in TNF-Tg mice. **(A)** A representative mouse heart WGA-stained section showed that the cross-sectional area of cardiomyocytes in TNF-Tg mice increased, and the cross-sectional area after HGWD treatment was smaller than that in the TNF-Tg group. Bar = 20 μm. **(B)** Quantification of the cross-sectional area of cardiomyocytes. Values are the mean plus or minus SD of 5 hearts in each group. *p < 0.05, compared with WT group; ^#^ *p* < 0.05, compared with TNF-Tg group.

**FIGURE 4:**
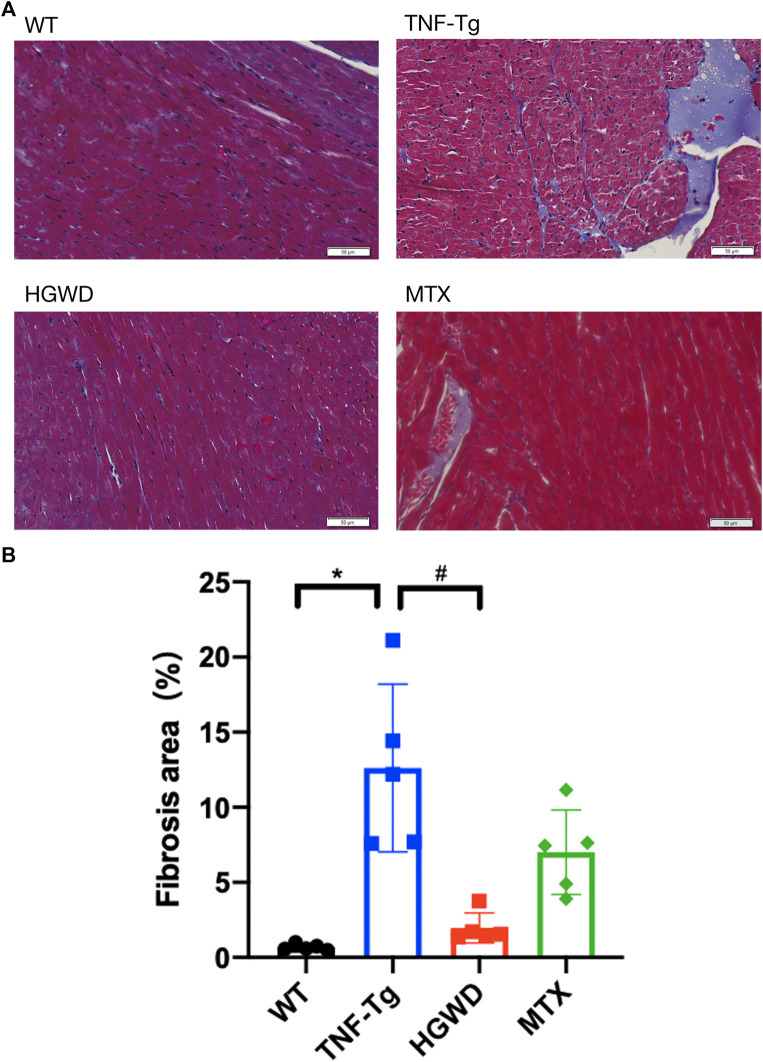
HGWD reduces myocardial fibrosis in TNF-Tg mice. **(A)** Representative Masson-stained sections of mouse heart showed obvious fibrosis, and the area of fibrosis decreased after HGWT treatment. Bar = 20 μm. **(B)** Quantification of the area of myocardial fibrosis. Values are the mean plus or minus SD of 5 hearts in each group. *p < 0.05, compared with WT group; ^#^ *p* < 0.05, compared with TNF-Tg group.

**FIGURE 5:**
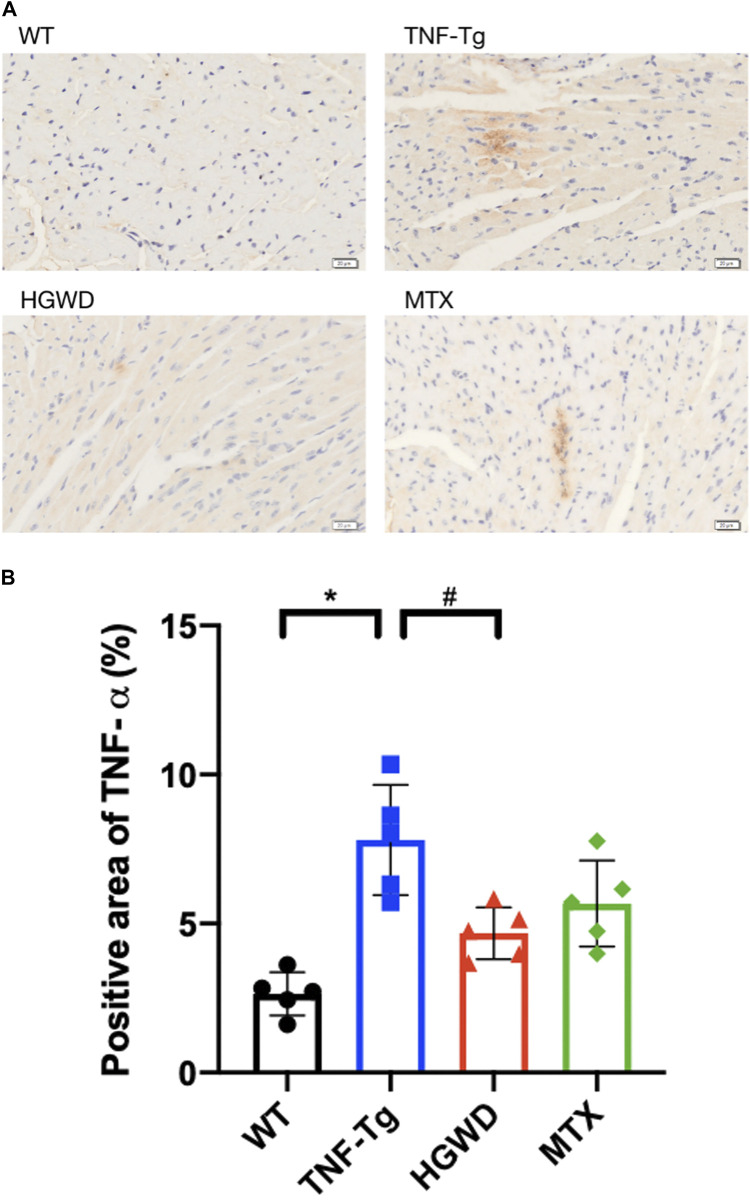
HGWD reduces the expression of TNF-*α* in myocardial tissue of TNF-Tg mice. **(A)** Representative mice heart immunohistochemical staining sections showed that the expression of TNF-*α* in TNF-Tg mice was increased, and after treatment, it was lower than that in the TNF-Tg group. Bar = 20 μm. **(B)** Quantification of the positive area of TNF-*α*. Values are the mean plus or minus SD of 5 hearts in each group. *p < 0.05, compared with WT group; ^#^ *p* < 0.05, compared with TNF-Tg group.

In addition, after staining the myocardial cell membrane of mouse myocardial tissue with WGA staining (Figure 3A), it was observed that the cross-section of myocardial cells of TNF-Tg mice (291.7 ± 62.70 μm^2^) was larger than that of the littermate group (162.4 ± 22.30 μm^2^) (Figure 3B). Treatment of HGWD can significantly reduce the inflammatory infiltration of myocardial tissue, and the cross-sectional area of myocardial cells was reduced, while the MTX administration group did not see a significant effect. Masson staining also showed that TNF-Tg mice had obvious myocardial fibrosis compared with the control group (Figures 4A, B). After the administration of HGWD, the area of myocardial fibrosis was significantly reduced. The area of myocardial fibrosis in the MTX administration group was also decreased, but it was not as effective as that. We also performed immunohistochemical staining of TNF-*α* on the hearts, and found more TNF-*α* expression in the heart of TNF-Tg mice, while the mice after taking HGWD decreased (Figure 5).

### Huangqi Guizhi Wuwu Decoction Alleviates Lung Damage in TNF-Tg Mice

**FIGURE 6:**
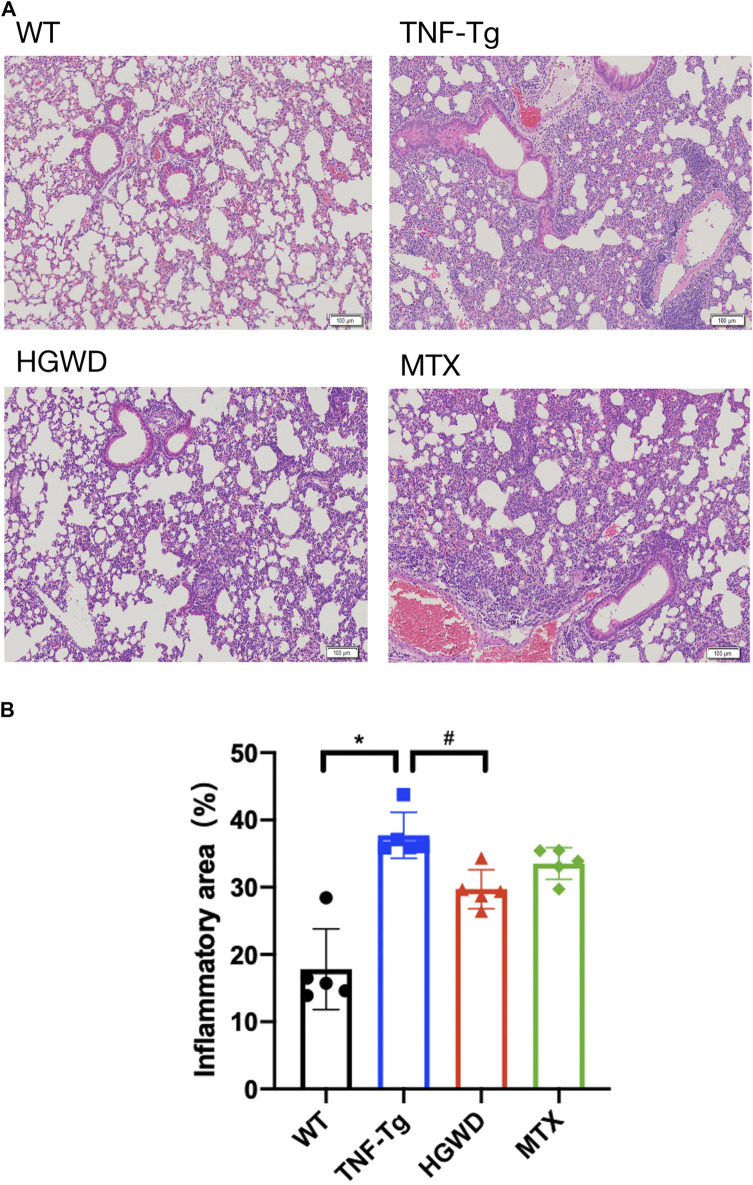
HGWD reduces lung inflammation infiltration in TNF-Tg mice. **(A)** TNF-Tg mice showed severe inflammatory infiltration near the lung bronchi, and the alveolar interval was significantly thickened. The HGWD-treated mice had reduced lung inflammation infiltration, and the alveolar interval was smaller than that in the TNF-Tg group. Bar = 100 μm. **(B)** Quantification of lung inflammation infiltration area. Values are the mean plus or minus SD of 5 lungs in each group. *p < 0.05, compared with WT group; ^#^ *p* < 0.05, compared with TNF-Tg group.

**FIGURE 7:**
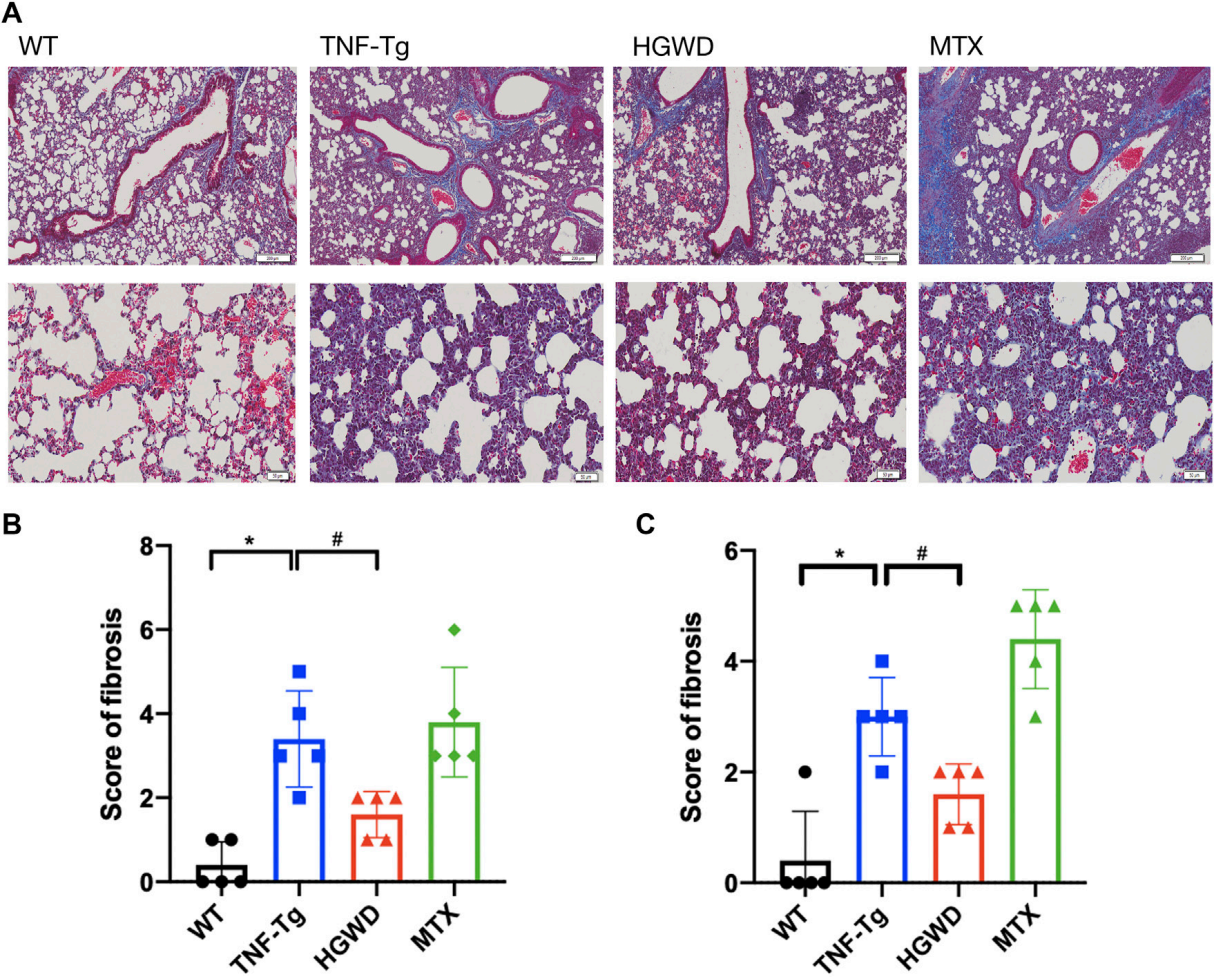
HGWD reduces lung fibrosis in TNF-Tg mice. **(A)** TNF-Tg mice have obvious fibrosis near the lung bronchus, and the fibrotic tissue is blue. The area of lung fibrosis in the mice treated with HGWD is reduced, and the lung fibrosis in the mice treated with MTX is aggravated. Bar = 200 μm or Bar = 50 μm. **(B)** Quantification of pulmonary peri bronchial fibrosis score. **(C)** Quantification of alveolar fibrosis score. Values are the mean plus or minus SD of 5 lungs in each group. *p < 0.05, compared with WT group; ^#^ *p* < 0.05, compared with TNF-Tg group.

Consistent with previous reports, we found that severe interstitial lung disease appeared in the lungs of TNF-Tg mice. The lungs of TNF-Tg mice were stained with HE, and the percentage of inflammatory area in lung tissue sections was quantified (Figures 6A, B). TNF-Tg mice had severe inflammatory infiltration near pulmonary bronchi, obvious thickening of the alveolar septum, and destruction of lung structure. HGWD treatment can reduce the inflammatory infiltration of the lungs of TNF-Tg mice, and reduce the thickening of the alveolar septum. However, the lung inflammation and structure of the TNF-Tg mice treated with MTX did not relieve. Similarly, using Masson staining and analysis with Ashcroft's (Ashcroft et al., 1988) lung fibrosis score, it was found that the lungs of TNF-Tg mice, including the vicinity of the bronchi and alveoli, had severe fibrous tissue proliferation (Figures 7A,B,C). Compared with the saline treatment group, the fibrosis around the bronchi and alveoli in the lungs of the HGWD treatment group was significantly relieved, and the fibrosis score decreased. The lung fibrosis of TNF-Tg mice in the MTX administration group was not reversed, but more severe tissue fibrosis appeared around the bronchus and alveoli.

## Discussion

The purpose of this study was to explore the pharmacological effects of HGWD in treating joint inflammation in TNF-Tg mice and its complicated cardiopulmonary diseases. The result shows that HGWD can significantly reduce joint synovial inflammation in TNF-Tg mice. At the same time, it also has a significant therapeutic effect on cardiopulmonary complications. HE, Masson, and WGA staining methods were used to process mouse joints, heart, and lungs, and statistically analyzed the results.

RA complicated with heart disease, including myocardial hypertrophy, myocardial fibrosis, and RA-ILD including pulmonary fibrosis and inflammatory infiltration, have been reported in other animal models of RA (Bell et al., 2020; Keith et al., 2012; Pironti et al., 2018; Redente et al., 2018). For the TNF-Tg model used in this study, It has been previously reported that TNF-Tg mice have lung lesions, including pulmonary inflammatory cell accumulation, pulmonary arteriole thickening, pulmonary fibrosis, and emphysema (Lundblad et al., 2005; Bell et al., 2018). Right ventricular hypertrophy has also been reported (Bell et al., 2019). In this study, we also found that TNF-Tg mice had pulmonary inflammatory cell aggregation and pulmonary fibrosis, as well as obvious thickening of the alveolar septum. In addition to myocardial hypertrophy, we also found significant inflammatory infiltration and increased fibrosis in the heart. It is suggested that TNF-TG mice can be used as a stable model for cardiopulmonary complications of rheumatoid arthritis in the study of RA complications.

MTX is a commonly used DMARDs for the treatment of RA. However, whether it has an effect on cardiovascular disease in patients with rheumatoid arthritis has always been of great interest to researchers. Controlling systemic inflammation and disease activity of RA to reduce the incidence of cardiovascular disease (CVD) in patients is a view that a large number of researchers currently agree on (Westlake et al., 2010; van Breukelen-van der Stoep et al., 2013; England et al., 2018; Atzeni et al., 2021). It is also possible to reduce CVD events in patients by improving activity functions and reducing the use of drugs that are harmful to the heart in the treatment of RA, such as non-steroidal anti-inflammatory drugs and glucocorticoids (van Breukelen-van der Stoep et al., 2013). And some studies believe that in the face of cardiovascular disease, MTX can participate in reducing the infarct size, inflammation, cardiac hypertrophy, and myocardial fibrosis after myocardial infarction by releasing adenosine (Asanuma et al., 2004; Headrick et al., 2013). However, it is controversial that some studies believe that this treatment has a small effect and is not enough to reduce the area of myocardial infarction or improve heart function (Maranhão et al., 2017; Dannenberg et al., 2021). In our study, it was also found that MTX-treated mice had a certain improvement in cardiac inflammation infiltration and cardiac fibrosis, but the effect was not significant, and MTX had no therapeutic effect on cardiomyocyte hypertrophy in TNF-Tg mice. Similarly, there are still many controversies regarding the use of MTX for RA-ILD patients (Fragoulis et al., 2019; Juge et al., 2021).

It was believed that the use of MTX was one of the causes of lung diseases (Sostman et al., 1976; McKendry and Dale, 1993; Conway et al., 2014; Salliot and van der Heijde, 2009; Carson et al., 1987; Hozumi et al., 2013) and also a risk factor for the occurrence and progression of RA-ILD (Gochuico et al., 2008; Conway et al., 2014; Hozumi et al., 2013). However, in recent years, clinical studies and meta-analysis have concluded that MTX has nothing to do with the development of RA-ILD (Juge et al., 2021; Ibfelt et al., 2021). Even MTX treatment may delay the onset of ILD (Kiely et al., 2019; Dawson et al., 2021). However, in our study, TNF-Tg mice given MTX did not show a reduction in lung fibrosis, but further aggravated fibrosis, including alveoli and bronchi. This is similar to Ohbayashi M’s finding that low-dose and long-term use of MTX can induce lung fibrosis in mice (Ohbayashi et al., 2010). Therefore, it is suggested that MTX is still controversial in the clinical treatment of patients with RA cardiopulmonary complications, and the medication should be used with caution in strict accordance with the drug indications.

Rheumatoid arthritis is a systemic inflammatory disorder that mainly affects the diarthrodial joint (Scott, 2010). The main pathogenesis is the continuous activation of immune cells, mainly T cells and macrophages, but also immune cells such as dendritic cells, B cells, and fibroblasts (Lin et al., 2020). Cytokines produced from many synovial cell groups are the core of the pathogenesis of rheumatoid arthritis, including a variety of cytokines TNF-*α*, IL-1 family, and IL-6 (Juge et al., 2020; McInnes et al., 2011). Overexpression of TNF-a in lung epithelial cells is also an important factor in the secondary interstitial pneumonia of rheumatoid arthritis (Salton et al., 2020). Therefore, the main treatment methods are to inhibit inflammation, relieve pain to relieve or reduce disease activity and improve joint function. HGWD has been used since the Han Dynasty and has a significant effect on the treatment of rheumatoid arthritis. Clinical studies have shown that patients treated with HGWD combined with western medicine can better relieve pain and joint swelling and improve joint mobility compared with patients in the western medicine control group (Liu et al., 2019; Yang et al., 2018). Animal experiments have found that HGWD can inhibit the expression of serum pro-inflammatory factors TNF-*α* and IL-1*β* in adjuvant arthritis rats and serum IL-20 and other inflammatory cytokines in CIA rats with collagen-induced arthritis (Liu et al., 2017; Xu et al., 2007), thereby alleviating chronic synovial inflammation in different rat models of arthritis. Network pharmacology also confirmed that TNF signaling pathway and IL-17 signaling pathway are important ways for HGWD to treat RA (Liu et al., 2020). This is consistent with our finding that the expression of TNF-*α* in the heart of mice treated with HGWD was reduced. In this study, the pathological conditions of TNF-Tg mice treated with HGWD were studied, and it was found that HGWD could not only reduce inflammation around the joints of TNF-Tg mice but also reduce the pathological changes in the heart and lungs.

Cardiopulmonary complications of RA seriously affect the quality of life of patients and increase the risk of death. However, the treatment of cardiopulmonary complications remains to be explored. We conducted the first study on the treatment of RA cardiopulmonary complications with plant extracts and found that HGWD has a significant effect on the treatment of cardiopulmonary pathological changes in TNF-Tg mice. It provides a theoretical basis and application basis for the prevention and treatment of RA complications and has important foundation and application value.

### Limitation

Due to the low reproductive rate of TNF-Tg mice and the small number of samples, it is difficult to apply multiple doses to study the dose-response relationship of HGWD. Therefore, in the experiment, we chose the dose of Chinese herbal medicine corresponding to the clinic. This dose was proved to be safe and effective in the previous study of CIA mice with gradient doses for the treatment of joint inflammation. The pharmacological effects of HGWD were not studied using gradient doses, which is one of the limitations of the study. This paper mainly observes the effect of HGWD on the joints, heart, and lungs of TNF-Tg mice from the pathological morphology. The pharmacological action and mechanism of HGWD cardiopulmonary protection will be studied in the future.

## Conclusion

HGWD decrease ankle joint inflammation in TNF-Tg mice, and reduced the pathological changes in the heart and lungs in TNF-Tg mice. HGWD could be a promising medicine for treating RA and RA cardiopulmonary complications.

## Data Availability

The original contributions presented in the study are included in the article/Supplementary Material, further inquiries can be directed to the corresponding authors.

## Abbreviations

CVD, Cardiovascular disease; CIA, Collagen-induced arthritis; DMARDs, Disease-modifying antirheumatic drugs; EDTA, Ethylene Diamine Tetraacetic Acid; HE, Hematoxylin and eosin; HGWD, Huangqi Guizhi Wuwu Decoction; MTX, methotrexate; RA, Rheumatoid arthritis; RA-ILD, Rheumatoid arthritis-related Interstitial lung disease; WT, Wild type; TNF-*α*, Tumor necrosis factor-*α*; ILD, interstitial lung disease; WGA, Wheat Germ Agglutinin.
