# Supplementary material for: Huangqi Guizhi Wuwu Decoction Improves Arthritis and Pathological Damage of Heart and Lung in TNF-Tg Mice
Source: Front Pharmacol. 2022 May 4;13:871481. doi: 10.3389/fphar.2022.871481 (PMC9114745; doi:10.3389/fphar.2022.871481)
Supplement: Supplementary file 1 [file Presentation1.PPTX]

## Slide 1
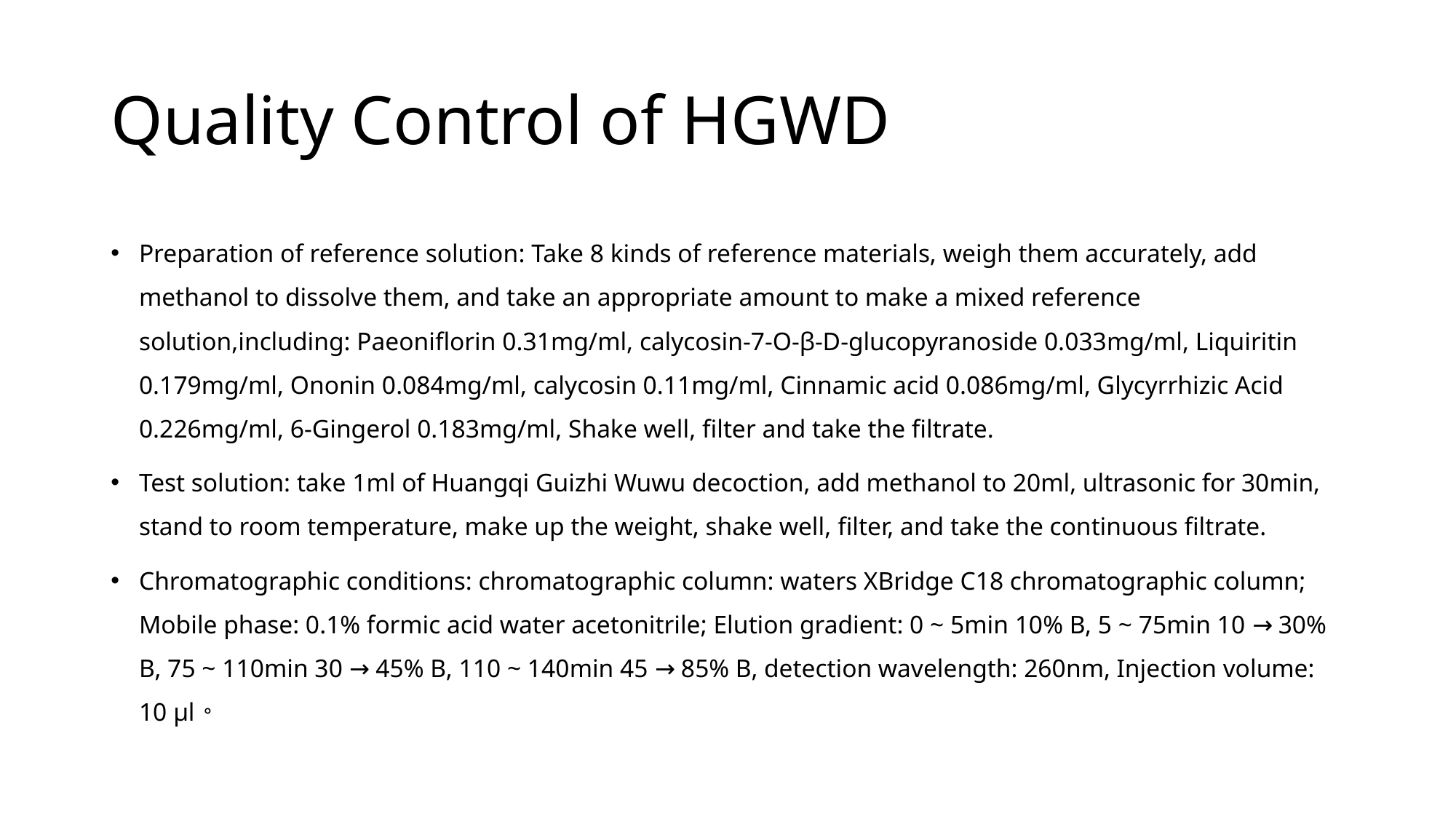

# Quality Control of HGWD
Preparation of reference solution: Take 8 kinds of reference materials, weigh them accurately, add methanol to dissolve them, and take an appropriate amount to make a mixed reference solution,including: Paeoniflorin 0.31mg/ml, calycosin-7-O-β-D-glucopyranoside 0.033mg/ml, Liquiritin 0.179mg/ml, Ononin 0.084mg/ml, calycosin 0.11mg/ml, Cinnamic acid 0.086mg/ml, Glycyrrhizic Acid 0.226mg/ml, 6-Gingerol 0.183mg/ml, Shake well, filter and take the filtrate.
Test solution: take 1ml of Huangqi Guizhi Wuwu decoction, add methanol to 20ml, ultrasonic for 30min, stand to room temperature, make up the weight, shake well, filter, and take the continuous filtrate.
Chromatographic conditions: chromatographic column: waters XBridge C18 chromatographic column; Mobile phase: 0.1% formic acid water acetonitrile; Elution gradient: 0 ~ 5min 10% B, 5 ~ 75min 10 → 30% B, 75 ~ 110min 30 → 45% B, 110 ~ 140min 45 → 85% B, detection wavelength: 260nm, Injection volume: 10 μl。

## Slide 2
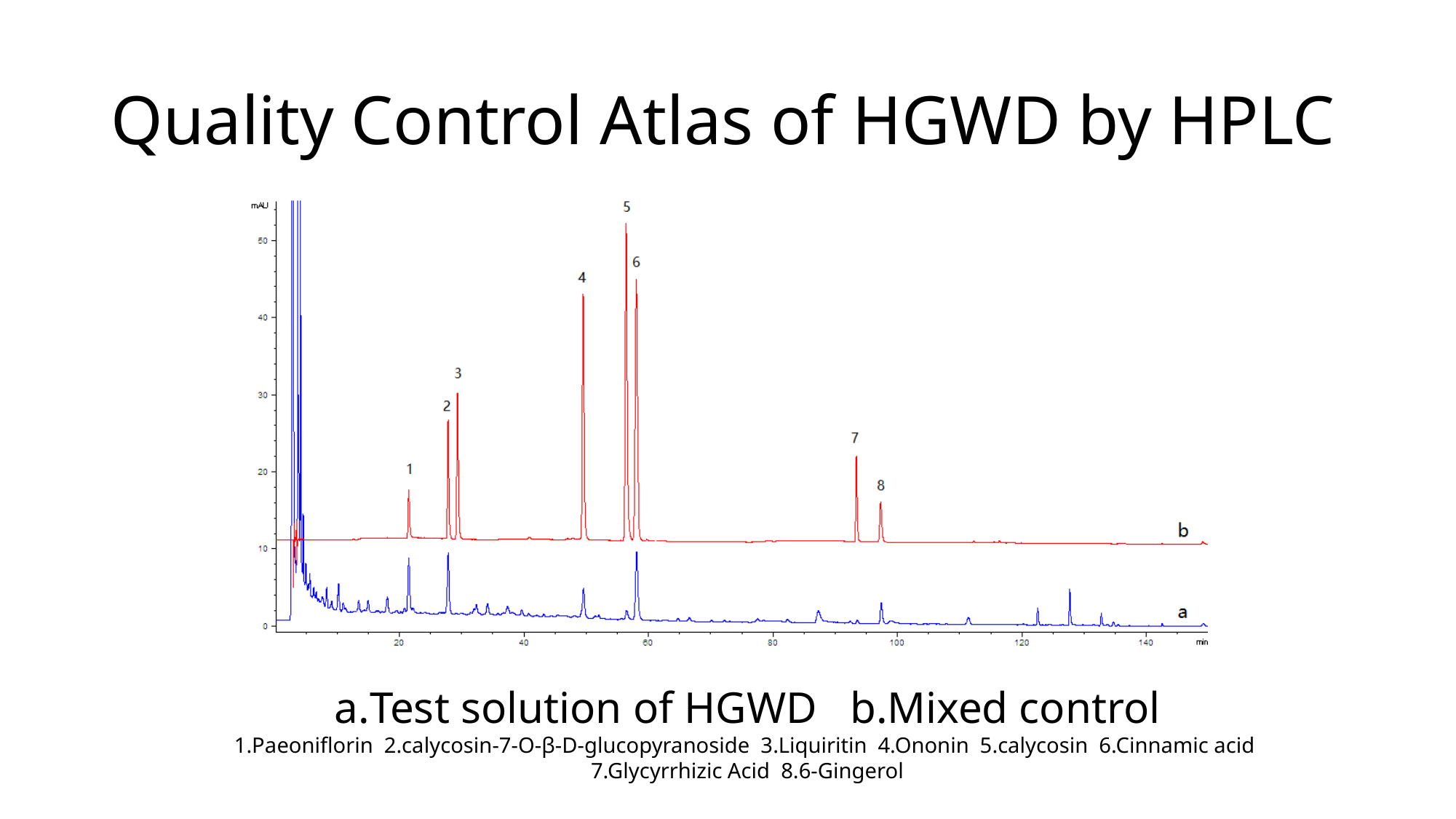

# Quality Control Atlas of HGWD by HPLC
a.Test solution of HGWD b.Mixed control
1.Paeoniflorin 2.calycosin-7-O-β-D-glucopyranoside 3.Liquiritin 4.Ononin 5.calycosin 6.Cinnamic acid 7.Glycyrrhizic Acid 8.6-Gingerol
